# Regular Communication Fulton County Medical Society

**Published:** 1916-06

**Authors:** 


### REGULAR MEETING OF THE FULTON COUNTY MEDICAL SOCIETY, JUNE 15, 1916.

Dr. Dunbar Roy read a paper on: "Some Statistics on Oto-Laryngological Diseases among Negroes."

Dr. Howard Hall agrees with Dr. Roy in reference to the necessity of room for free passage of air through the nose and throat. He thinks that proper ventilation here avoids, in a great measure, sinus infections.

Dr. R. B. Ridley, Jr., stated that while his clinical experience has not been so great as that of Dr. Roy, that in his work he has found the observation of Dr. Roy to be true in the great majority of cases. Free ventilation, in the negro race, he thinks, accounts for the absence of sinus infections. In his experience he has found that 80% of all negro patients who go to clinics with nose and throat trouble are luetic and yield only to specific treatment. He considers that the high arch and the broad face account for the lack of spurs and deviations of the septum in the negro race. However, there are very few Caucasians who do not have some deviation of the septum. The chronic running ear is not noticed as frequently now as it was sometime ago, which he thinks is due to the fact that adenoids and diseased tonsils are removed early and thereby preventing further complications. Dr. Ridley recalled that in a meeting of the Ga. State Medical Association in 1900, Dr. A. W. Calhoun stated that adenoids were not frequently found in negroes. This, he thinks, accounts for the prevalent opinion that they are infrequent in the negro race.

Dr. E. L. Griffin asked that Dr. Roy explain the following conditions in his closing remarks: (1) Earache; (2) Ringing in the ears; (3) Running ears.

Dr. M. T. Davis stated that he considers one of the frequent causes of tinnitus aurium to be high blood pressure. He also reported that he had used vaccines in the treatment of otitis media.

Dr. R. B. Ridley, Jr., stated in reply to the question of Dr. Roy in reference to diphtheria among negroes, that he had seen very little diphtheria among them.

Dr. Walpole Brewer reported that in his six months' experience in one of the negro wards of a New York Hospital he found most all of the patients there to be light colored mulattoes. He thinks these mulattoes have less resistance to tuberculosis, syphilis and to all other diseases than the full black negro and the white man. In his experience there he thinks this is the class of negro most generally affected with troubles of this kind.

In closing, Dr. Roy stated that tinnitus aurium is frequently due to high blood pressure. For earache, Dr. Roy recommended the following: (1) Drop chloroform on a piece of cotton and place in ear; (2) 10 grains of orthoform to one-half ounce of liquid albolene and make an emulsion. Warm, shake well, and put a few drops in the ear; (3) Onion poultice. Boil onions thoroughly and mix with meal and apply to ear while hot.

Dr. F. C. Thrash read a paper on "Cryptic Bacteriemias." This paper appears in this issue of the Journal.

In discussing the paper of Dr. Thrash, Dr. C. W. Gould, reported that he had excellent success with blood cultures in using solid culture media. The media is first liquified by heating and then cooled down to 45 degrees C. and the blood put in and plates made. He described the appearance of colonies of streptococcus, pneumococcus and streptococcus veridans on the blood agar plates, stating that this was a very excellent method of identifying these organisms.

Dr. Allen H. Bunce stated that the paper of Dr. Thrash was very valuable from the fact that it called attention to the importance of not overlooking bacteriemias after the primary focus of infection had healed and disappeared. Ordinarily blood cultures are made only upon patients where there is a focus infection present, except, of course, in cases of suspected typhoid fever. However, Dr. Thrash called attention to the presence of a possible bacteriemias in a great many other conditions. In reference to the technique of making blood cultures, Dr. Bunce stated that as a routine measure, he preferred citrated glucose bouillon. He thinks that an elaborate technique is not necessary to prevent contaminations, as one experienced in laboratory work can easily avoid this by proper precautions. Of course, it is possible to get a staphylococcus as a contamination from the skin and this should always be guarded against as carefully as possible. He states that it is his custom to obtain a large quantity of blood, at least 10 to 20 c. c. and to use a large quantity of culture media, as he thinks that a great many negative results are obtained on account of not obtaining a sufficient amount of blood or not using a large enough quantity of media.

Dr. M. T. Davis stated that he wished to especially commend Dr. Thrash for bringing this subject to the attention of the Society. He stated that blood cultures had been of value to him in his work. He reported a case of a young boy in which he diagnosed septicemia with malignant endo-carditis. However, a consultant gave a different opinion with a more favorable prognosis. Blood culture made by Dr. Bunce showed the streptococcus. The patient subsequently died as Dr. Davis had predicted that he would.

